# Perinatal mental health research: priorities from a neurodiverse sample in the United Kingdom

**DOI:** 10.1007/s00737-026-01734-x

**Published:** 2026-06-22

**Authors:** Alexandra Lautarescu, Siofra Heraty, Chloe Johnson, Amber Ruigrok, Eliza Eaton, Tessel Bazelmans, Julie Sigurdardottir, Victoria Ratti, Holly Stone, Jenna Anderson, Ayesha Javed, Shivali Mahtani, Alex Lau-Zhu, Debbie M. Smith

**Affiliations:** 1 Department of Forensic and Neurodevelopmental Sciences, Institute of Psychiatry, Psychology, and Neuroscience, King’s College London, London, UK; 2 Bloomfield Hospital, Rathfarnham, Dublin, Ireland; 3 Department of Psychology, Institute of Psychiatry, Psychology, and Neuroscience, King’s College London, London, UK; 4 Division of Psychology and Mental Health, University of Manchester, Manchester, UK; 5 University of Cambridge, Autism Research Centre, Cambridge, UK; 6 Centre for Brain and Cognitive Development, Birkbeck, University of London, London, UK; 7 Department of Biostatistics and Health Informatics, King’s College London, London, UK; 8 WANDA Community Focus Group, London, UK; 9 Department of Experimental Psychology, University of Oxford, Oxford, UK; 10 Child and Adolescent Mental Health Services, Oxford Health NHS Foundation Trust, Oxford, UK; 11 Department of Psychology, Canterbury Christ Church University, Canterbury, UK

**Keywords:** Perinatal mental health, Participatory research, Research priorities, Neurodiversity, Mixed-methods

## Abstract

**Purpose:**

The aim of this study is to better understand perinatal mental health research priorities of neurotypical and neurodivergent people.

**Methods:**

A co-designed mixed-methods survey was conducted as part of the Wellbeing Across Neurotypes: Depression and Anxiety (WANDA) study. 709 participants in the United Kingdom (UK) quantitatively rated and ranked research topics within perinatal mental health, with 253 also providing qualitative data.

**Results:**

The top research priorities were (1) interventions and (2) diagnosis for perinatal mental health problems for birthing people and (3) how these impact parent-infant relationships. Qualitative data revealed additional topics of importance including healthcare experiences, trauma, and neurodiversity.

**Conslusions:**

These results suggest complex, multilevel barriers to accessing diagnosis and support in the UK.

**Supplementary Information:**

The online version contains supplementary material available at https://doi.org/10.1007/s00737-026-01734-x.

## Introduction

Mental health difficulties impact over 25% of those who give birth (Office of Health Improvement & Disparities 2025; Smythe et al. 2022), with long-standing negative effects on the wellbeing of parent(s) and children (Howard and Khalifeh 2020). Neurodivergent people (e.g., autism, attention deficit hyperactivity disorder, ADHD; including those who self-identify) are at increased risk (Hampton et al. 2022; Murray et al. 2022). In the United Kingdom (UK), recent concerns were raised about the state of maternity services and the resulting psychological impact (APPG 2024). In this context, it is essential to understand perinatal mental health research priorities of women and birthing people, as well as whether these differ between neurodivergent and neurotypical people. While some have reported on the research priorities of neurodivergent participants (Pellicano et al. 2014), and separately on perinatal research priorities in the general population (Mossinger et al. 2023), to our knowledge, this is the first community-based participatory priority-setting exercise within perinatal mental health in the UK to include a neurodiverse sample.

## Materials and methods

### The WANDA study

The Wellbeing Across Neurotypes: Depression and Anxiety (WANDA, https://osf.io/xg3vc) study aims to better understand perinatal mental health risk and resilience factors and how these relate to neurodivergence. WANDA is co-produced by a diverse team of multi-disciplinary researchers and lived experience experts, with community members paid for their time following NIHR guidelines. The survey (See Supplementary Material and https://osf.io/xg3vc/files/ngvw9) was iteratively developed through regular focus groups, email communication, and collaborative online documents. Ethical approval was obtained from King's College London Research Ethics Committee (HR/DP-22/23-36808, HR/DP-22/23-38899). All participants provided written informed consent. Qualitative data was included only for participants who have provided consent for the publication of anonymous quotations.

### Recruitment

Participants were eligible if they were aged ≥ 16 years, lived in the UK, and had recent experience of pregnancy (i.e., were currently pregnant and/or had been pregnant in the last 5 years). Data collection took place August 2023 to January 2024. We used a convenience sample method intended to reach a variety of viewpoints and to over-sample for neurodivergence and history of poor mental health. Online flyers were disseminated via social media (e.g., Facebook, Instagram, Twitter/X), organisations (e.g., Maternity Autism Research Group, ADHD Adult UK, Dyspraxia UK, PACT Southwark, Best Beginnings, Birthing Trauma Association), forums (e.g., Mumsnet, Netmums), and through snowball sampling (i.e., asking participants to share the survey within their networks). Printed flyers were posted in public areas (e.g., notice boards, community centres, libraries, cafes, university buildings). The survey was hosted on the Qualtrics online platform and took approximately 20 min to complete. As a thank you for their contributions, participants were entered into a prize draw with a chance to win an Amazon voucher (£10-£100). Bot responses were identified and excluded using a comprehensive strategy (See Supplementary Material and https://osf.io/ad4ny).

### Survey

This analysis is based on a subset of questions from the WANDA survey. Participants were shown a list of 15 research topics focused on perinatal mental health and co-developed by the WANDA team based on the World Health Organisation Action Plan 2013–2030 (see Supplement Table S1). Participants were asked to select “the top 5 topics that you think are most important for researchers to focus on”. For these 5 choices, participants were then asked to “rank these topics in the order of importance to you, with 1 being most important”. Lastly, participants were provided with an open-text response box about whether there were “any other topics or questions that you think researchers should focus on”. Demographics were collected, including history of mental health (depression, anxiety, other) and neurodevelopmental conditions, NDC (autism, ADHD, other NDC), with answer options: “No, I have never been diagnosed with this”, “Yes, I have been diagnosed with this”, “Yes/Likely yes, I have never received a diagnosis but this applies to me/I self-identify with this” and “Maybe, I think this may apply to me but I am not sure”.

### Statistical analysis

Quantitative data was cleaned and analysed using R (version 4.3.2) and the full code is available at https://github.com/AleLautarescu/WANDA_Priorities. Sample categorisation by neurodivergence was determined through focus group discussions. Main results are reported for the whole sample and for the neurodivergent subsample (“NDC subsample”, those responding “Yes” or “Likely Yes” to autism, ADHD, and/or Other NDC). Additional results are reported by further categorising participants into 7 non-mutually exclusive subgroups: by condition (autism, ADHD, other NDC) and response type: “Yes” (including one or more “Yes” or “Likely Yes”), “Maybe” (but excluding those with any “Yes” or “Likely Yes” response), or No NDC (No to all three).

Qualitative data from the open-ended question were analysed following an approach based on Krippendorff’s content analysis (Krippendorff 1989). Answers were read by two researchers independently (AL, SH) to exclude non-valid entries (e.g., “do not want to answer”), achieving consensus on the dataset to be analysed. They independently identified preliminary codes, which were compared and refined through discussion to develop a final codebook. AL and CJ independently sorted the coding units (sub-sentences/excerpts) into the agreed codes and achieved consensus through a series of discussions. Codes were discussed with another researcher (DMS) for agreement purposes. Frequency codes were calculated.

## Results

### Quantitative data

709 participants were included (median age 34 years, range 21–53). Participants were oversampled for neurodivergence, including autism (between 12.1% “yes/likely yes” and 23% also including “maybe”), ADHD (14.7–27.2%), and other neurodevelopmental conditions (11.6–14.8%), and for history of poor mental health, including depression (52.5–60.8%), anxiety (59.8–71.5%), and other mental health conditions (21.4–26.8%). Many participants had several diagnoses and/or intersectional identities.

Most participants identified as women (96.9%), heterosexual (82.5%), white (90.8%), were university educated (82.2%), and in a relationship (95.8%). 18.9% of participants were pregnant at the time of completing the survey and the remainder had been pregnant in the previous 5 years. 32% participants had been pregnant during COVID-19 pandemic lockdowns.

**Fig. 1:**
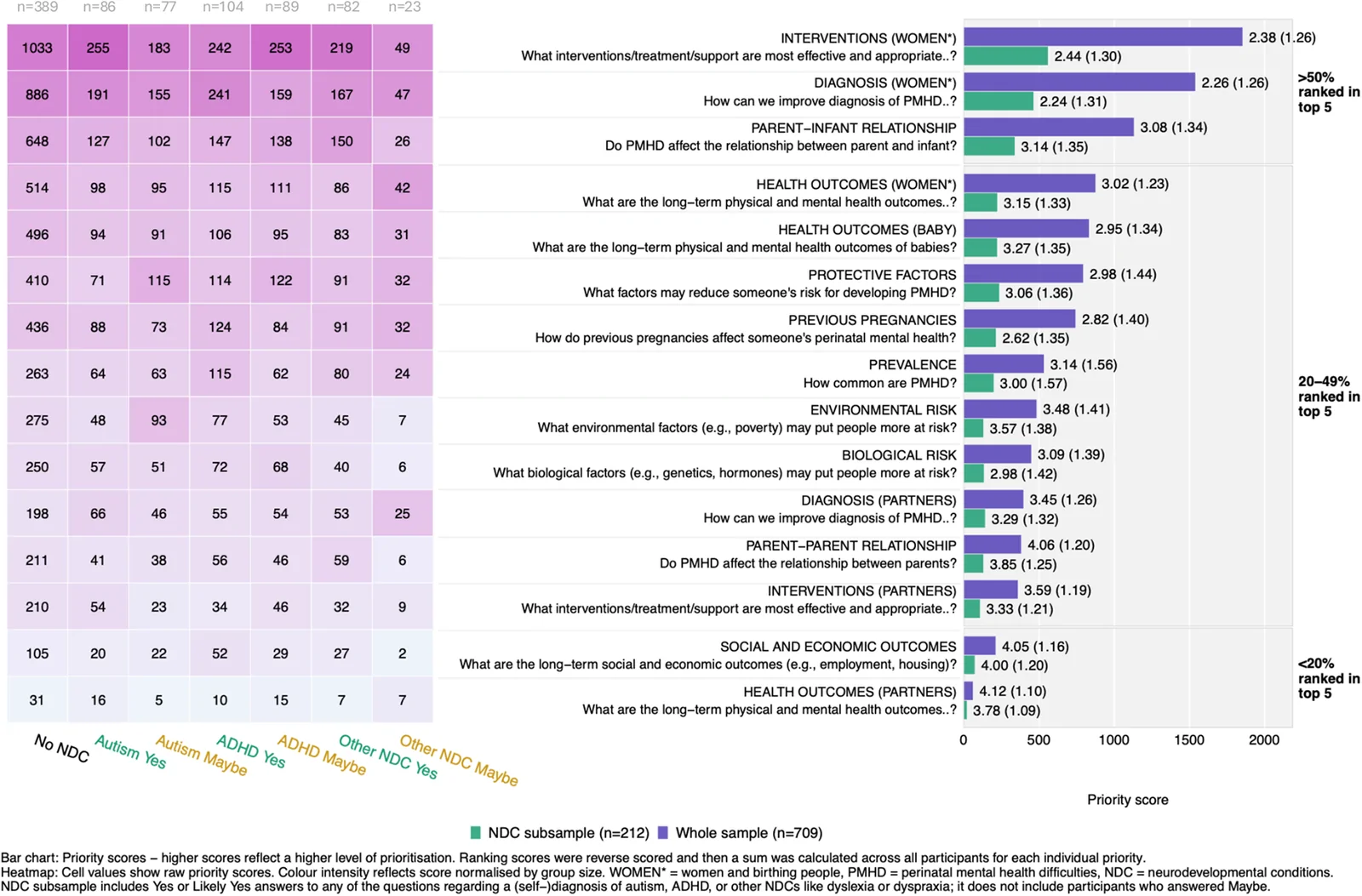
Perinatal mental health research priorities across neurotypes. Left: heatmap displaying priority scores for each subgroup, with colour intensity reflecting scores normalised by group size to allow visual comparison across groups. Right: bar chart for the whole sample and NDC subsample, displaying mean rank and standard deviation, ordered by priority scores. Priority scores reflect the sum of reverse-scored rankings across participants (higher score = higher priority). Mean rank reflects the average ranking per participant (1 = highest possible rank, 5 = lowest possible rank)

In the whole sample and the NDC subsample, the top research priorities identified by participants included interventions and diagnoses for women and birthing people, as well as parent-infant relationships (see Fig. 1). The bottom research priorities included health outcomes for partners and social/economic outcomes of poor perinatal mental health. There are small differences in the order of priority scores between the whole sample and the NDC subsample, as well as between the NDC subgroups (See Fig. 1 and Table S2).

### Qualitative data

Qualitative data included *n* = 253 participants, as *n* = 3 participants did not consent to their qualitative data being used and *n* = 453 participants did not provide a (valid) response to this question. A summary of the resulting themes and corresponding quotes is presented in Table 1, with reporting including neurodivergence. Despite participants being asked to list “any *other* topics or questions …”, many wrote about topics already in the list provided, adding examples of research questions (e.g., for “prevalence” - “The prevalence of mental health struggles and the specific struggles of autistic or ADHD birthing people”), suggesting specific sub-topics (e.g., “Family/friend support of the pregnant person” as a protective factor), or adding additional detail regarding the importance of a topic (e.g., “The hardest and most traumatic part for me was my experience of the postnatal ward - it would be useful to include this in your work”, as environmental risk). To capture this nuance, some of the existing topics were subdivided into separate codes (Table 1 Section A. Existing themes). Key additional themes (Table 1 Section B) included healthcare experiences, perinatal trauma, birth experiences, and socio-economic circumstances. Neurodivergent participants also highlighted the importance of researching neurodivergence, including specific experiences (e.g., “Does autistic meltdown in pregnancy affect baby”), associations between neurodiversity and physical health (e.g., “Any link to hyperemesis and neurodiversity”) or differences in how neurodivergent people are treated in perinatal settings (e.g., “I felt that the process is tailored to neurotypical people and left me feeling anxious because I felt there was a lot of stuff I was expected to know / do without being told”). Some participants highlighted the lack of research into perinatal mental health in neurodivergent women, with focus often being on infant outcomes (“Anything I tried to find out about only led me to things about ‘how to prevent autism during pregnancy’”) (See Table 1 and Supplementary Table S3).

**Table 1:** Qualitative data regarding research priorities

Theme		*N* Whole Sample (Maybe NDC/NDC Subsample)	Example quote
**Section A. Existing themes**: Themes that match those presented as part of the quantitative ranking question, presented in the priority order as per Fig. 1.			
*Please note that some themes were subdivided into additional codes.*			
Interventions for women*	**Generic comments**	40(2/15)	“What type of therapy works best”, “How GPs can support”, “Types of support available”
	**Medication**	4(0/3)	“More research into safety of medication.”, “Medicine in pregnancy and real life risk of their use”
Diagnosis for women*		17(2/10)	“Identifying mental health in women at the earliest opportunity”, “Misdiagnosis”
Parent-infant relationship		4(0/2)	“Healthy parental attachment”, “Impact of birth trauma on (…) parent-child bonding”
Health outcomes for women*		3(1/0)	“Mental health & therefore the impact of public policy on mother health outcomes”
Health outcomes for baby		5(0/2)	“Does autistic meltdown in pregnancy affect baby”, “Impact of public policy on baby health outcomes”
Protective factors	**Generic comments**	8(1/4)	“What can women do to prevent mental health issues during the pregnancy”
	**Social networks & support**	7(2/2)	“Social networks - having friends with babies.”, “Family/friend support of the pregnant person”
	**Positive healthcare exp.**	6(1/3)	“The impact of continuous care from a midwife on mental health”, “More structured antenatal care”
Previous pregnancies	**Generic comments**	1(0/0)	“Previous pregnancies and how this can affect mental health, perinatal care and baby health”
	**Loss**	18(2/3)	“Mental health impact of recurrent miscarriage.”, “Cumulative impact of pregnancy loss”
	**Infertility**	6(1/1)	“The impact of IVF and infertility on mental health”, “How difficulty conceiving can affect perinatal health”
Prevalence		7(0/4)	“How common postnatal depression is”, “Prevalence of PTSD after traumatic birth”
Environmental risk	**Generic comments**	3(0/2)	“Environmental risk; whether or not internal or external factors are more important/how they interact”
	**Negative healthcare experiences**	33(4/12)	“A lot of my deteriorating mental health was as a result of how I was treated on the NHS”, “Women are often treated as vessels rather than people”, “The role of healthcare professionals in creating birth trauma”, “Obstetric violence”, “Obstetric gaslighting”
	**Baby’s health and behaviour**	10(2/2)	“Risk for parents of premature/NICU babies.”, “Impact of baby sleep and or crying (purple crying/colic crying) on parental mental health”, “Infant feeding in relation to mental health”
	**Parental relationship**	4(1/0)	“How parents’ relationship can affect the mental health during pregnancy”, “Domestic abuse and pregnancy”, “The link of a partners mental health on the mother’s mental health”
	**COVID-19**	8(1/1)	“The effect of covid lock down on mother’s mental health”, “Covids impact on first time motherhood”
	**Prejudice**,** stigma**,** and expectations**	20(2/7)	“Effects of race on the maternity experience”, “The impact of social standards and pressure puts in women to remain quiet about their struggles …”, “Feeling judged.”, “how body size impacts how you are treated …”
Biological risk	**Generic comments**	2(0/0)	“Understanding connections between certain weeks/trimesters and mental health symptoms”
	**Physical health**	18(1/7)	“Hyperemesis and mental health”, “Mental health recovery of birth tears, postpartum hemmorage.”
	**Pre-existing mental health**	5(2/1)	“Previous experiences of mental health difficulties and if they increase peoples risk .”
Diagnosis for partners		0	-
Parent-parent relationship		3(0/1)	“Parent-parent relationship after birth”
Interventions for partners		5(0/2)	“There needs to be more support in place for partners of women who experience perinatal mental illnesses.”
Social and economic outcomes		0	-
Health outcomes for partners		4(1/1)	“Perinatal mental health impact in (…) their partners when their baby is in NICU.”
Section B. New themes^4^: Additional themes that did not match those presented as part of the quantitative ranking question			
Healthcare experiences - non-directional		27(2/13)	“How a midwife’s work style affect a pregnant woman’s experiences”; “Gaining informed consent and communication styles”, “How NHS care impacts the mental wellbeing of mothers”
Trauma		27(5/10)	“Impact of birth trauma on parental wellbeing”, “Cptsd”, “The birth (...) is the most traumatising experience someone can go through if it doesn’t go well. It can impact for years”
Birth experiences		12(5/1)	“The birth is important”, “Benefits of elective c sections - leading to thoughts on whether they could be use more or discouraged less to protect mental health” “The birth can either give the mother incredible powers or take power and strength from her”
Neurodiversity		24(0/23)	“Supporting neurodivergent people better in pregnancy and labour”, “Any links to hyperemesis and neurodiversity”, “Hospital care of neurodivergent patients”, “The specific struggles of autistic or ADHD birthing people”
Access to services/support/information		43(4/19)	“The long queues for support. I’ve waited 9 months for pregnancy support but haven’t received it it will now be postnatal support I receive”, “More public information about postpartum stage”, “Knowing where to get help”, “I can say that the waiting list in my area for perinatal care seems longer than pregnancy:”
Socio-economic circumstances		13(0/4)	“The link between perinatal mental health difficulties and systemic inequalities”, “How low maternity pay impacts mental health”, “Socio-economic barriers to accessing support”
Breastfeeding		8(2/2)	“How ending breastfeeding affects mental health”, “Impact of difficulties with breastfeeding on post-partum mood/anxiety”
Hormones		3(0/0)	“The profound effect of hormones post birthing”
Workplace		5(0/2)	“How the workplace can react or support those struggling”, “Impact of work and stress onto pregnancy”

1 - Existing themes are presented in the same order as Fig. 1, with topics subdivided into additional codes where deemed necessary (e.g., the “interventions” theme is subdivided into “generic comments” and “medication”)

2 - “No NDC” = those who answered “No” to all questions about neurodevelopmental conditions, “Maybe NDC” = those who answered “Maybe” to at least one question about neurodevelopmental conditions, and did not answer “Yes” to any, “NDC” = those who answered “Yes” or “Likely yes” to at least one question about neurodevelopmental conditions. Counts represent the number of responses that fit under a code

3 – women* = “for women and birthing people”

4 - Themes with only a small number of responses were included in the supplementary material: generic comments about risk, prenatal/postnatal mental health, partner’s health, and other relationships; comments that do not fit under a theme

All qualitative data are included in the Supplementary Material

## Discussion

This is the first study reporting on perinatal mental health research priorities of neurodiverse people. While many neurodivergent people advocated for more neurodiversity-focused and inclusive research, others identified priorities that mirrored those of neurotypical participants. Across our sample, participants emphasized the need for research into (1) effective interventions and support for birthing people experiencing poor perinatal mental health, and (2) improved diagnosis of perinatal mental health conditions. These priorities align with calls for better identification and management of maternal mental disorders (Howard and Khalifeh 2020) and with evidence of complex, multilevel barriers to accessing support in the UK (Smith et al. 2019).

Undiagnosed and/or untreated perinatal mental health problems can have long term adverse impacts on women/birthing people, as well as on their child(ren) (Rogers et al. 2020). Participants in our study highlighted the need for more research into the impact of poor perinatal mental health on (3) parent-infant relationships and health outcomes of both (4) women and (5) their children.

Highlighting the value of mixed-methods approaches, the qualitative data revealed priorities that extend beyond those captured by the forced-choice ranking exercise. Birth experiences and healthcare interactions emerged as key priorities for future research, often described as interconnected (“the role of healthcare professionals in creating birth trauma”, “women are often treated as vessels rather than people”) and echoing findings from the recent reports on birth trauma (APPG 2024). Participants emphasized the need to understand how these could serve as either risk or protective factors for perinatal mental health (“The birth can either give the mother incredible powers or take power and strength from her”). For some, qualitative responses reflected deeply personal experiences, highlighting how individual circumstances shape research priorities, including those related to identities that are intersectional and/or historically excluded from perinatal mental health research (e.g., “experiences of neurodivergent black British caribbean women”). To address the limitations in our study and ensure that research and services better reflect the needs of *all* families, it is imperative that further research prioritises the inclusion of people from underrepresented socioeconomic and ethnic backgrounds (Miller et al. 2024), whose voices are critical given participants’ emphasis on systemic inequalities and barriers to care. Future studies should be based on neurodiversity-affirmative frameworks (Heraty et al. 2023) and investigate priorities of those with multiple neurodivergent identities, as well as those who are uncertain about or still seeking a diagnosis.

## Supplementary Information

Below is the link to the electronic supplementary material.

Supplementary Material 1

## Data Availability

The study survey and analysis code for the quantitative analysis are provided in full as OSF and GitHub links. The qualitative data is shared in full as part of the Appendix, for all participants who have given consent for this. We do not have participant consent to share any of the quantitative data at participant level.
