# Supplementary material for: Perinatal mental health research: priorities from a neurodiverse sample in the United Kingdom
Source: Arch Womens Ment Health. 2026 Jun 22;29(4):99. doi: 10.1007/s00737-026-01734-x (PMC13287293; doi:10.1007/s00737-026-01734-x)
Supplement: Supplementary file 1 — Supplementary Material 1 [file 737_2026_1734_MOESM1_ESM.docx]

**Supplementary Material**

Table S1. Research priorities from the [STUDY NAME REDACTED FOR DOUBLE-ANONYMOUS PEER REVIEW] survey and correspondence to WHO action plan indicators for Objective 4

| **WHO Action plan indicators^1^** | **Research priorities included in the** [STUDY NAME REDACTED FOR DOUBLE-ANONYMOUS PEER REVIEW]  **survey** |
| --- | --- |
| “The prevalence of mental disorders” | “**Prevalence**: How common are perinatal mental health difficulties” |
|  | “**Diagnosis (for women and birthing people):** How can we improve diagnosis of perinatal mental health difficulties for women and birthing people?” |
|  | “**Diagnosis (for partners**): How can we improve diagnosis of perinatal mental health difficulties for partners” |
| “Identification of major risk factors … for mental health and wellbeing” | “**Biological risk**: What biological factors (e.g., genetics, hormones) may put people more at risk for developing perinatal mental health difficulties?” |
|  | “**Environmental risk:** What environmental factors (e.g., poverty) may put people more at risk for developing perinatal mental health difficulties?” |
|  | “**Previous pregnancies**: How do previous pregnancies affect someone’s perinatal mental health?” |
| “Identification of major protective factors … for mental health and wellbeing” | “**Protective factors:** What factors may reduce someone’s risk for developing perinatal mental health difficulties?” |
| “Interventions and services” | “**Interventions (for women and birthing people)**: What interventions/treatment/support are most effective and appropriate for women and birthing people who experience perinatal mental health difficulties?” |
|  | “**Interventions (for partners):** What interventions/treatment/support are most effective and appropriate for partners who experience perinatal mental health difficulties?” |
| “Health outcome data” | “**Health outcomes (for women and birthing people):** What are the long-term physical and mental health outcomes of women and birthing people who experience perinatal mental health difficulties?” |
|  | “**Health outcomes (for partners):** What are the long-term physical and mental health outcomes of partners who experience mental health difficulties?” |
|  | “**Health outcomes (baby):** What are the long-term physical and mental health outcomes of babies whose parent(s) experience mental health difficulties?” |
| “Social and economic outcome data” | “**Social and economic outcomes**: What are the long-term social and economic outcomes of people who experience perinatal mental health difficulties (e.g., employment, housing)?” |
| “Coverage of policies and legislation” | *NA – Determined to be outside of the scope of this study by the* [STUDY NAME REDACTED FOR DOUBLE-ANONYMOUS PEER REVIEW] co-creation *groups* |
| *Not included in WHO Action Plan but determined to be a key priority by the* [STUDY NAME REDACTED FOR DOUBLE-ANONYMOUS PEER REVIEW] co-creation *groups* | “**Parent-infant relationship:** Do perinatal mental health difficulties affect the relationship between parent and infant?” |
|  | “**Parent-parent relationship:** Do perinatal mental health difficulties affect the relationship between parents?” |

1- Extracted from [World Health Organisation (WHO) Action Plan 2013-2030](https://iris.who.int/bitstream/handle/10665/345301/9789240031029-eng.pdf): Objective 4 (To strengthen information systems, evidence and research for mental health)

Table S2. Community research priorities for perinatal mental health research, with the order determined by priority score for the whole sample

| **% ranked in top 5** | **Topic** | **Whole sample n = 709** | | | | **NDC subsample n=212**^1^ | | | |
| --- | --- | --- | --- | --- | --- | --- | --- | --- | --- |
|  |  | **N (%) top 5** | **Mean rank (SD)** | **Priority score**^2^ | **Priority order** | **N (%) top 5** | **Mean rank (SD)** | **Priority score**^2^ | **Priority order** |
| >50% | **Interventions (for women and birthing people):**  What interventions/treatment/support are most effective and appropriate for women and birthing people who experience perinatal mental health difficulties?” | 512 (72.2%) | 2.38 (1.26) | 1853 | 1 | 157  (74%) | 2.44 (1.3) | 559 | 1 |
|  | **Diagnosis (for women and birthing people):** How can we improve diagnosis of perinatal mental health difficulties for women and birthing people? | 411 (58%) | 2.26 (1.26) | 1539 | 2 | 123  (58%) | 2.24 (1.31) | 463 | 2 |
|  | **Parent-infant relationship:** Do perinatal mental health difficulties affect the relationship between parent and infant? | 387 (54.6%) | 3.08 (1.34) | 1131 | 3 | 118  (55.7%) | 3.14  (1.35) | 338 | 3 |
| 20-49% | **Health outcomes (for women and birthing people):** What are the long-term physical and mental health outcomes of women and birthing people who experience perinatal mental health difficulties? | 294 (41.5%) | 3.02 (1.23) | 875 | 4 | 78  (36.8%) | 3.15  (1.33) | 222^3^ | 5^4^ |
|  | **Health outcomes (baby):** What are the long-term physical and mental health outcomes of babies whose parent(s) experience mental health difficulties? | 273 (38.5%) | 2.95 (1.34) | 832 | 5 | 81  (38.2%) | 3.27  (1.35) | 221^3^ | 6^4^ |
|  | **Protective factors:** What factors may reduce someone’s risk for developing perinatal mental health difficulties? | 263 (37.1%) | 2.98 (1.44) | 793 | 6 | 80  (37.7%) | 3.06  (1.36) | 235^3^ | 4^4^ |
|  | **Previous pregnancies:** How do previous pregnancies affect someone’s perinatal mental health? | 233 (32.9%) | 2.82 (1.40) | 742 | 7 | 63  (29.7%) | 2.62 (1.35) | 213 | 7 |
|  | **Prevalence:** How common are perinatal mental health difficulties? | 186 (26.2%) | 3.14 (1.56) | 532 | 8 | 66  (31.1%) | 3 (1.57) | 198 | 8 |
|  | **Environmental risk:** What environmental factors (e.g., poverty) may put people more at risk for developing perinatal mental health difficulties? | 192 (27.1%) | 3.48 (1.41) | 483 | 9 | 53     (25%) | 3.57 (1.38) | 129^3^ | 12^4^ |
|  | **Biological risk:** What biological factors (e.g., genetics, hormones) may put people more at risk for developing perinatal mental health difficulties? | 154 (21.7%) | 3.09 (1.39) | 448 | 10 | 44  (20.8%) | 2.98 (1.42) | 133 | 10 |
|  | **Diagnosis (for partners):** How can we improve diagnosis of perinatal mental health difficulties for partners? | 155 (21.9%) | 3.45 (1.26) | 396 | 11 | 52  (24.5%) | 3.29 (1.32) | 141^3^ | 9^4^ |
|  | **Parent-parent relationship:** Do perinatal mental health difficulties affect the relationship between parents? | 196 (27.6%) | 4.06 (1.20) | 381 | 12 | 60  (28.3%) | 3.85 (1.25) | 129^3^ | 11^4^ |
|  | **Interventions (for partners):** What interventions/treatment/support are most effective and appropriate for partners who experience perinatal mental health difficulties?” | 149 (21%) | 3.59 (1.19) | 359 | 13 | 40  (18.9%) | 3.33 (1.21) | 107 | 13 |
| <20% | **Social and economic outcomes:** What are the long-term social and economic outcomes of people who experience perinatal mental health difficulties (e.g., employment, housing)? | 108 (15.2%) | 4.05 (1.16) | 211 | 14 | 36  (17%) | 4 (1.2) | 72 | 14 |
|  | **Health outcomes (for partners):** What are the long-term physical and mental health outcomes of partners who experience mental health difficulties? | 32 (4.5%) | 4.12 (1.10) | 60 | 15 | 9  (4.2%) | 3.78  (1.09) | 20 | 15 |

SD = Standard Deviation

1 - The NDC subsample includes all participants who answered “Yes” or “Likely yes” to any of the questions regarding a diagnosis or self-diagnosis of autism, ADHD, or other neurodevelopmental conditions like dyslexia or dyspraxia; it does not include participants who answered “Maybe”

2 - Priority scores are calculated so that higher scores reflect a higher level of prioritisation. Ranking scores were reverse scored (i.e., 5=1, 4=2, 3=3, 2=4, 5=1) and then a sum was calculated across all participants for each of the individual priorities

3 - Indicates topics where the priority score order is different between the whole sample and the neurodivergent subsample; cells where the priority score is higher in the neurodivergent subsample than the whole sample are represented in dark grey; where lower, in light grey.

4 – as above, for “priority order”

Table S3. Qualitative data

| **Type** | **Main theme** | **Sub-theme (where relevant)** | **Participant quote** |
| --- | --- | --- | --- |
| E | Prevalence | | Prevalence in both the birther and their partner |
|  |  |  | Personality and prévenance of post natal depression |
|  |  |  | … how common postnatal depression is… |
|  |  |  | … prevalence of pmdd after giving birth (a correlation highlighted in many forums) |
|  |  |  | Prevalence of.. PTSD after traumatic birth |
|  |  |  | The prevalence of mental health struggles and the specific struggles of autistic of ADHD birthing people … |
|  |  |  | Prevalence of mental health issues in BAME women during their pregnancies, compared to non-BAME |
| E | Diagnosis (women and birthing people) | | … How can the health service better identify (...) women who experience perinatal mental health difficulties … |
|  |  |  | How to improve recognition of potentially higher risk individuals, and those that may be neurodiverse and not know it. Maybe have some sort of screening tool that can highlight potential conditions such as ADHD and ASD that increase the risk of adverse mental health in an otherwise seemingly low-risk person, especially since conditions like these can make it more difficult for someone to recognise they need help and actually seek out help (even more so if we throw RSD in the mix, too) … |
|  |  |  | Identifying mental health in women at the earliest opportunity |
|  |  |  | Diagnosis and signs and symptoms to look for |
|  |  |  | … so that people can be identified and diagnosed accurately |
|  |  |  | … could GPs reach out to assess difficulties soon after birth |
|  |  |  | Misdiagnosis |
|  |  |  | … the problems need to be better understood first |
|  |  |  | Diagnosis is still important too |
|  |  |  | How to identify women & birthing people who may not have insight into their own mental health difficulties |
|  |  |  | Correct risk assessment and full evaluation not paper tigers |
|  |  |  | … diagnosis of mental health … in the initial postpartum period |
|  |  |  | Would adding some screening questions to the booking visit questionnaire that all pregnant women do at their first appointment have any beneficial affect? |
|  |  |  | Postnatal depression and how it can appear (...) how someone can recognise it in themselves |
|  |  |  | Are all care providers trained in identifying signs of mental health problems |
|  |  |  | … timings of diagnoses … |
|  |  |  | I think more attention should be paid to maternal mental health issues, sometimes a lot of women do not express their psychological problems |
| E | Diagnosis (partners) | | NA |
| E | Biological risk | Generic comments | … both elements of risk factors … *(n.b. environmental and biological risk)* |
|  |  |  | Understanding connections between certain weeks/trimesters and mental health symptoms |
|  |  | Physical health | The impacts of the physical elements of pregnancy and the postnatal period on mental health e.g. diastasis recti |
|  |  |  | Hyperemesis and mental health |
|  |  |  | … how physical symptoms arise in pregnancy |
|  |  |  | How might factors such as chronic illness effect mental health in pregnancy |
|  |  |  | The link between particular pregnancy health conditions and mental health issues |
|  |  |  | … severe endometriosis |
|  |  |  | Any link to Hyperemesis and Neurodiversity? |
|  |  |  | Chronic illness, such as autoimmune, and pregnancy |
|  |  |  | … was not helpful. Not was the continual idea that suffering was just normal ie hardly being able to walk (...) Nor was any impact from the physical problems ever discuss linking to mental health despite the fact that I could hardly walk.. |
|  |  |  | How are parent-infants that are affected with unusual congenital anomalies that do not fit into a standard pathway of care impacted on their health and eduction outcomes. |
|  |  |  | How physical conditions can have an effect on mental health. I myself suffered with Hyperemisis Gravidarum (HG) in three pregnancies. (...) I was terrified and no one told me I had a condition and it wasn’t being treated correctly. My second and third pregnancies I had the same condition but I was more prepared, especially by the third. (...) The anxiety of knowing I was probably going to suffer from HG again was terrifying but I have a fantastic partner and family unit to turn to for support which 100% got me through. |
|  |  |  | Effects of conditions in pregnancy and mental health |
|  |  |  | Links with physical wellbeing and ability to exercise or perform tasks at the same level as pre pregnancy - what impact this has on mental health |
|  |  |  | Hyperemesis gravidarum and it's impact on mental health |
|  |  |  | How employers support women with severe sickness and fatigue during pregnancy. How women manage this whilst working e.g whether or not to disclose before 12 weeks, what happens when sickness is only a part of the day so would prefer not to be signed off sick all together but need accommodations made … |
|  |  |  | Mental health recovery of birth tears, postpartum hemmorage, long hospital and at home recovery’s. More help for those in severe pain post birth |
|  |  |  | … I’ve looked into it alot now about the awful feelings I got on let down of milk and its quite common but not spoken about … |
|  |  | Pre-existing mental health | How previous mental health can be impacted … |
|  |  |  | … I had a history of PTSD due to sexual violence, a previous suicide attempt and bulimia. I was worried how the pregnancy would affect my mental health and that I could become very unwell again |
|  |  |  | Tokophobia, especially for those who are not pregnant but want to have a child but the fear is too great … |
|  |  |  | Previous trauma not necessarily related to pregnancy or birth |
|  |  |  | Perhaps previous experiences of mental health difficulties and if they increase peoples risk of perinatal MH problems, particularly experiencing ‘severe and enduring’ eg psychosis type disorders and if so which mental health difficulties eg depression, anxiety lead to increased risk |
| E | Environmental risk | Generic comments | Environmental risk; whether or not internal or external factors are more important/how they interact |
|  |  |  | … both elements of risk factors … *(n.b. environmental and biological risk)* |
|  |  |  | Bonding between parents and child especially in a situation such as a pandemic or large crisis such as war |
|  |  | Negative healthcare experiences | How obstetric violence affect birthing people |
|  |  |  | Experiences in healthcare including racism |
|  |  |  | … is *(n.b. patient relapse)* due to lack of care from mental health services |
|  |  |  | … the hardest and most traumatic part for me was my experience of the postnatal ward - it would be useful to include this in your work. |
|  |  |  | … Women are often secondary in their own pregnancies - what is best for the baby, and treated as vessels rather than people. We have names and do not want to delegated to simply 'mum' … |
|  |  |  | Quickly having lots of information thrown at you is difficult to process and can result in additional stress unnecessarily,  An example is the recent changes to induction guidelines which are not being communicated effectively. I was told of this change at my last midwife appointment before giving birth and this added significant stress to my plans for birth that could have easily been avoided by communicating earlier in my pregnancy. |
|  |  |  | … I should also point out I did have a couple of unrelated appointments last year where the person doing the appointment flagged potential depression to my GP (unknown to me until I went through my history when looking into potential ADHD) and yet I’d have never known despite it being raised on two separate occasions by two different places - nobody has been touch to check in, and it just seems like nobody from the GP practice has even seen it … |
|  |  |  | The role of healthcare professionals in creating birth trauma |
|  |  |  | The impact of poor health care in pregnancy i.e. lots of scare inducing language from health professionals, inappropriate use of statistics around risk, making comments not founded in science |
|  |  |  | There needs to be more protection in order to avoid trauma during hospital stays/Birthing experience |
|  |  |  | Do unwanted interventions or coercion in labour increase risk of perinatal mental distress |
|  |  |  | …  I asked for mental health support to safeguard me and my child and hopefully make it possible for me to access maternity care and have a natural labour. They refused to see me as not actively suicidal and the clinical psychology team in primary care I was referred to urgently did not offer me an appointment until four weeks after giving birth. So pretty terrible... |
|  |  |  | How midwives and hospital staff can make a (...) negative impact on pregnant women |
|  |  |  | Obstetric violence, as this has a huge correlation with mental health issues but is very under researched and misunderstood. |
|  |  |  | In particular, GPs do not tend to know much about what medication for mental health is safe in pregnancy and in my experience, And others I know, were just told to stop it for the pregnancy instead of being given the risks etc. |
|  |  |  | … I had to wait two months and was given CBT which was inappropriate. The practitioner had no experience with pregnancy (...) So the CBT approach of reframing worries simply wasn’t appropriate. And didn’t help me. |
|  |  |  | Through my experiences I have reflected on one thing which I feel has made my pregnancy and miscarriage experiences more traumatic. I can point to a number of occasions where I wasn’t listened to and could have been offered help and support earlier. Those moments stick with me today … |
|  |  |  | My midwife appointments were all fairly brief and went over the same questions every time. It also want easy to bring up my real fears or anxiety around things like tearing. |
|  |  |  | Supporting midwives to shift away from the attitude that 'you have a healthy baby/you got through the birth' of your last baby and therefore that you must be okay. |
|  |  |  | Impact of denying skin to skin on mental health of mother and baby (for me, not holding my babies at birth or days afterwards was the worst part of it all) |
|  |  |  | I felt that the process is tailored to neurotypical people and left me feeling anxious because I felt there was a lot of stuff I was expected to know / do without being told. And things like the 1-10 pain question just doesn’t make sense to me - and I think I had to go through more pain & trauma because of this. |
|  |  |  | Being told different advice and feeling judged during pregnancy was not helpful. Not was the continual idea that suffering was just normal ie hardly being able to walk. It made you feel insecure asking about things mental not physical when already physical problems were dealt with with such variation and any distress taken as for granted. It did not encourage reaching out for mental health help. Nor was any impact from the physical problems ever discuss linking to mental health despite the fact I could hardly walk and was in lockdown in poor circumstances |
|  |  |  | … My midwife did not refer me when I raised them *(n.b. concerns)* |
|  |  |  | Obstetric violence (...) obstetric gaslighting |
|  |  |  | … no one told me I had a condition and it wasn’t being treated correctly *(n.b. hyperemesis gravidarum) …* |
|  |  |  | … the right resources (my midwife appointments were conducted in temporary office structures without access to a toilet, which was unacceptable and disrespectful...) |
|  |  |  | There is no knowledge of this *(n.b. tokophobia)* or understanding by nhs staff or support. You are not taken seriously my medical staff. Also how to get support pre pregnancy and early pregnancy as Gp receptionists all said pregnancy was nothing to do with the Gp you have to self refer to midwives but how do you do that when this is your first time you don’t know Gp staff didn’t know then when you do get in touch they say to send in your paper work at 10-12 weeks but no access to any support before that |
|  |  |  | The complete lack of any support for people who are considered "not at risk" because they don't have the usual socio-economic factors. Despite having lost my previous baby and being looked after in pregnancy by the bereavement team, once the baby was safely born there was an assumption that I no longer required any mental health support despite the fact that the stress of the pregnancy had essentially destroyed my mental health and that of my partner. When I asked for help I was told that I didn't qualify for any support because I wasn't self-harming or suicidal and that if I wanted therapy I could pay for it myself. I wanted practical support like what's offered by Home Start but wasn't allowed this because of my postcode. |
|  |  |  | … A lot of my deteriorating mental health was as a result of how I was treated on the NHS after my loss |
|  |  |  | Mental health issues caused by (...) poor care during pregnancy, birth and postpartum |
|  |  |  | The effects of dehuminising language such as “birthing bodies” on pregnant women’s mental health, particularly when used by health professionals/organisations |
|  |  |  | … medical staff,  especially on the maternity wards, are, in dangerously high number,  people with no empathy, unprofessional, not knowing and applying mothers and babies rights. I know that from many experiences that i hear every day, not only mine. Unfortunately,  a mother that is not strongly informed before birth, is almost sure a victim of uninformed consent, " if you don't specifically say "No" to a procedure, it means that you agree with it" … |
|  |  |  | … Midwifes seem to just wanna rush you to discharge. Also how  if you are an experienced parent like my self with 5 children how they make you feel like they know best when actually no from personal experience I may no better. Yes I might not no the medical ins and outs but I'm pretty confident on whats normal or not when it comes to my baby. |
|  |  | Baby’s health and behaviour | risk for parents of premature/NICU babies and interventions |
|  |  |  | … Impact of baby sleep and or crying (purple crying/ colic crying) on parental mental health … |
|  |  |  | Perinatal mental health impact in people and their partners when their baby is in NICU and life after NICU |
|  |  |  | More aftercare for parents who's babies where admitted to neonatal care, more so when they get home |
|  |  |  | Impact of prematurity/nicu (...) Impact of denying skin to skin on mental health of mother (...) (for me, not holding my babies at birth or days afterwards was the worst part of it all) |
|  |  |  | How are parent-infants that are affected with unusual congenital anomalies that do not fit into a standard pathway of care impacted on their health and eduction outcomes. |
|  |  |  | Infant feeding in relation to mental health |
|  |  |  | The impact of sleep deprivation on parents with infants |
|  |  |  | Impact of baby health concerns during pregnancy, and how that affects behaviours in terms of caring for your newborn |
|  |  |  | Supporting the mental health of mothers and fathers whose babies end up needing extra support following birth |
|  |  |  | How preterm birth affects mental health |
|  |  | Parental relationship | How parents’ relationships can affect the mental health during pregnancy and how this therefore affects the baby |
|  |  |  | I think domestic abuse in pregnancy is an important topic, I didn’t realise until it happened to me that this is the most common time people experience abuse, I think there needs to be more research into it, why it happens, why we stay around for it, identifying it as healthcare professionals, identifying it as the pregnant women, and knowing where to get help. There needs to be better support for anyone suffering abuse, especially at such a vulnerable time being pregnant |
|  |  |  | Domestic abuse and pregnancy |
|  |  |  | The link if a partners mental health on the mother's mental health. |
|  |  | COVID-19 | impacts now compared to covid |
|  |  |  | Covids impact on first time motherhood |
|  |  |  | The effect of covid lock down on mother’s mental health |
|  |  |  | Not was any impact from the physical problems ever discuss linking to mental health despite the fact I could hardly walk and was in lockdown in poor circumstances |
|  |  |  | Impact of covid on birthing people’s mental health |
|  |  |  | My sister has a baby at the start of covid and the difference in our experiences are huge |
|  |  |  | I do think covid had a large impact as I think my overall experience and possibly things that happened would not have if we had not been in the pandemic |
|  |  | Prejudice, stigma, and expectations | reduce stigma |
|  |  |  | Experiences in healthcare including racism and support while being treated |
|  |  |  | … Women are often secondary in their own pregnancies - what is best for the baby, and treated as vessels rather than people. We have names and do not want to delegated to simply 'mum' … |
|  |  |  | … Expectations of parenthood & the impact on parent, child & partner |
|  |  |  | Attitudes of health professionals towards perinatal mental health and how they conceptualise this |
|  |  |  | Effects of race on the maternity experience |
|  |  |  | The attitude towards mental health from professionals, the media |
|  |  |  | Reducing stigma on parents getting mh support |
|  |  |  | … the Impact social standards and pressure puts in women to remain quiet about their struggles with mental health in and after pregnancy. |
|  |  |  | … feeling judged during pregnancy was not helpful. Not was the continual idea that suffering was just normal  … |
|  |  |  | Why is there still a stigma attached to mental health during pregnancy/birth/post birth? |
|  |  |  | …  I'm also concerned with the mental health problems hypnobirthing courses and books can lead to as a particular birth type tends to be sold as best and it is pushed that it is down to the efforts and preparation the birthing parent puts in to achieve that. They avoid discussion of or downplay the fact that things around births are sometimes out of our hands which can lead to parents feeling like failures or as if they haven't done it right. |
|  |  |  | … There is a blame culture at the moment - if baby doesn’t sleep well, that’s mum’s fault for lack of sleep training or routine, breastfeeding, picking baby up too often etc … |
|  |  |  | Social media influences on expectations and pressure |
|  |  |  | How perinatal mental health difficulties intersect with race/ethnicity - looking specifically at the lived experiences of neurodivergent black British caribbean women. It’s something I would really like to hear more about. |
|  |  |  | - Prevalence of mental health issues in BAME women during their pregnancies, compared to non-BAME.   - Comparison of how both groups are supported by the healthcare system with respect to their mental health |
|  |  |  | The effects of dehuminising language such as 'birthing bodies' on pregnant women's mental health, particularly when used by health professionals/organisations. |
|  |  |  | Stigma and why people don’t access support and how to change this |
|  |  |  | How body size impacts how you are treated by medical professionals during pregnancy and how this impacts mental health. |
|  |  |  | normalising these feelings |
| E | Previous pregnancies | Generic comments | Previous pregnancies (...) and how this can affect mental health, perinatal care and baby health |
|  |  | Loss | How does previous pregnancy loss affect mental health |
|  |  |  | Past miscarriages and how this can affect mental health, perinatal care and baby health |
|  |  |  | Cummulative impact of pregnancy loss |
|  |  |  | Miscarriage effects on mental health |
|  |  |  | Mental health impact of recurrent miscarriage and interventions to manage this during the next pregnancy |
|  |  |  | The practitioner had no experience with pregnancy and my overwhelming worry following two losses was that my baby would die inside me and I'd have to give birth to a dead child. |
|  |  |  | Through my experiences I have reflected on one thing which I feel has made my pregnancy and miscarriage experiences more traumati*c (n.b. poor healthcare experiences)* |
|  |  |  | Follow ups for people experiencing pregnancy following a loss |
|  |  |  | How prior miscarriages (...) can affect perinatal health |
|  |  |  | I think we do very little care or research in case of pregnancy loss. I feel that it is important to the care of subsequent successful pregnancy that prior loses impact on mental health be properly considered. Post natal psychosis and post natal depression when there is no living infant at simply not cared for at all. |
|  |  |  | My first experience ended with a termination because I was young, I was terrified and no one told me I had a condition and it wasn’t being treated correctly *(n.b. hyperemesis gravidarum).* (...)  the termination always played on my mind and the feeling of guilt I felt whilst being pregnant was overwhelming. |
|  |  |  | … Despite having lost my previous baby and being looked after in pregnancy by the bereavement team, once the baby was safely born there was an assumption that I no longer required any mental health support despite the fact that the stress of the pregnancy had essentially destroyed my mental health and that of my partner … |
|  |  |  | … The impact of multiple miscarriages on mental health |
|  |  |  | Understanding the impact of miscarriages (...) for women who do go on to have a successful pregnancy is rarely explored. It feels like you should get over all the problems once you have a baby but I know many feel a lot of complicated grief once a baby arrives. |
|  |  |  | Treatment and care of people, and (the lack of) emotional support given to people who suffer a miscarriage in the early weeks of pregnancy (before 2 months) |
|  |  |  | Additional support in subsequent pregnancies after loss. There is so little support on the NHS. Charities pick up the shortfall in care. There needs to be more focus on prevention of deteriorating mental health. Alot of my deteriorating mental health was as a result of how i was treated on the NHS after my loss |
|  |  |  | Providing more support to women who have experienced a loss |
|  |  |  | Exercise support so you feel safe to exercise even after previous miscarriages. |
|  |  | Infertility | How does infertility affect mental health. |
|  |  |  | Impact of fertility treatment on mental health |
|  |  |  | Fertility (...) effects on mental health |
|  |  |  | The impact of IVF and infertility on mental health. We had IVF for both of our pregnancies and I have severe endometriosis. |
|  |  |  | How (...) difficulty conceiving can affect perinatal health |
|  |  |  | Understanding the impact of (...) infertility treatment for women who do go on to have a successful pregnancy is rarely explored. It feels like you should get over all the problems once you have a baby but I know many feel a lot of complicated grief once a baby arrives. |
| E | Protective factors | Generic comments | … Breastfeeding and access to lactation consultants and feeding community groups (LLL) |
|  |  |  | when should we start providing people with tools to support their mental health? and awareness? and reduce stigma? |
|  |  |  | … Impact of having practical support post-partum on recovery (and mental health). |
|  |  |  | There needs to be more protection in order to avoid trauma during hospital stays/Birthing experience |
|  |  |  | … It would also be helpful to know how to protect mental health before becoming pregnant and interventions related to this. |
|  |  |  | Why is it some women who experience traumatic pregnancies/labour do not end up with  perinatal mental difficulties. |
|  |  |  | What can women do to prevent mental health issues during the pregnancy |
|  |  |  | … How employers and local authorities can work with families by adopting  sensible policy. |
|  |  | Social networks and support | Social networks - having friends with babies, access to children's centres etc. … |
|  |  |  | … Never to leave vulnerable pregnant women alone without support of loved ones. What affect has that had on mental health and future outcomes? … |
|  |  |  | Family/friend support of the pregnant person |
|  |  |  | … but I have a fantastic partner and family unit to turn to for support which 100% got me through. |
|  |  |  | How good levels of support affect mental health during and after pregnancy |
|  |  |  | Peer support and community visibility of parents |
|  |  |  | I … have very supportive friends and family |
|  |  | Positive healthcare experiences | Informing mothers more widely and further in advance of issues / process / changes to process during pregnancy and birth. |
|  |  |  | How midwives and hospital staff can make a positive (...) impact on pregnant women |
|  |  |  | The impact of continuous care from a midwife on mental health … |
|  |  |  | … On the flip side there were moments where people made hard experiences much more bearable because of the time they took to explain and listen to me (this wasn’t more than 5 mins max). Those times do not stick out as traumatic  … |
|  |  |  | Whether more structured antenatal care would improve birth and therefore mental health. |
|  |  |  | Correct risk assessment and full evaluation not paper tigers |
|  |  |  | … I had an incredible experience with services after giving birth to my second child … |
| E | Interventions (women and birthing people) | Generic comments | when should we start providing people with tools to support their mental health ? and awareness ? … |
|  |  |  | how to help people experincing perinatal mental health problems through pregnancy and beyond |
|  |  |  | … How can the health service better (...) support women who experience perinatal mental health difficulties. What changes to the health service can provide better care and support to women. (...). There is no point identifying factors and rates of difficulties if there is nothing in place to change that |
|  |  |  | … interventions |
|  |  |  | … dealing with it *(n.b. mental health)* as a matter of urgency |
|  |  |  | The importance of support (...) for post-birth |
|  |  |  | … how to better support neurodiverse parents … |
|  |  |  | … how GPs can support *(n.b. mental health)* |
|  |  |  | Types of support available |
|  |  |  | … It would also be helpful to know how to protect mental health before becoming pregnant and interventions related to this |
|  |  |  | What type of therapy works best |
|  |  |  | … Perinatal support needs to be longer and does that reduce effects of mental illnesses etc by giving more resources and time |
|  |  |  | … interventions to manage this *(n.b. mental health impact of recurrent miscarriage)* during the next pregnancy |
|  |  |  | How to access help more effectively and in a timely manner. How to not to put more pressure on the one seeking help … |
|  |  |  | How to improve interventions after the birth to minimise duration of mental ill health. |
|  |  |  | Impact on further pregnancies and how to support women (...) through it |
|  |  |  | Providing effective help quickly. I had to wait two months and was given CBT which was inappropriate. The practitioner had no experience with pregnancy and my overwhelming worry following two losses was that my baby would die inside me and I'd have to give birth to a dead child. So the CBT approach of reframing worries simply wasn't appropriate. And didn't help me. |
|  |  |  | I think the interventions are really important too … |
|  |  |  | This may link with intervention but thinking about trauma informed care within maternity settings would have so much value - not only in regards to trauma survivors but as these principals would likely also allow for more culturally sensitive care and better care for neurodiverse individual. |
|  |  |  | More aftercare for parents who's babies where admitted to neonatal care, more so when they get home |
|  |  |  | How mental health issues are treated/dealt with and followed up |
|  |  |  | There needs to be better support for anyone suffering abuse, especially at such a vulnerable time being pregnant |
|  |  |  | Improving services for women experiencing mental illness in pregnancy. It's a scary place to be. |
|  |  |  | Support (...) of mental health (...) in the initial postpartum period |
|  |  |  | Understanding that treatment can be varied, so CBT may work for some … |
|  |  |  | How to better support women throughout pregnancy and birth |
|  |  |  | More research into (...) treatments for mental health for pregnant and postpartum people |
|  |  |  | … support for PTSD after traumatic birth |
|  |  |  | I have often wondered about exercise specifically as an intervention for mental health difficulties during pregnancy … |
|  |  |  | Postnatal depression and (...) how it can be treated |
|  |  |  | Are all care providers trained in (...) how to support/signpost birthing individuals … |
|  |  |  | The relationship between different neurotypes and support (...) is it effective if not what can we do to support various neurotypes |
|  |  |  | Treatment and care of people … |
|  |  |  | I long term rather than short term approaches to mental health are useful. The difference between being seen 6 times in 6 weeks vs 6 times in 6 months. By the time folk are seen for mental health they maybe highly motivated to do homework and therfore longer term support might work better than intensive cbt with zero follow up. |
|  |  |  | … There needs to be more focus on prevention of deteriorating mental health … |
|  |  |  | … More help for those in severe Pain post birth |
|  |  |  | Providing more support to women who have experienced a loss |
|  |  |  | Supporting the mental health of mothers (...) whose babies end up needing extra support following birth |
|  |  |  | Especially support for breastfeeding and the affect it has on your mental health as you feel so pressured into doing it but not actually given any support for your mental health part of it. |
|  |  |  | It’s important to remember that women who may not have suffered/experienced mental before pregnancy/birth can experience really awful times during this and the support needs to be explored more about what is on offer. |
|  |  | Medication | … In particular, GPs do not tend to know much about what medication for mental health is safe in pregnancy and in my experience, And others I know, were just told to stop it for the pregnancy instead of being given the risks etc. |
|  |  |  | Medicine in pregnancy and real life risk of their use |
|  |  |  | Understanding that treatment can be varied so (...) medications *(n.b. may work)* for others |
|  |  |  | More research into safety of medication (...) for mental health for pregnant and postpartum people |
| E | Interventions (partners) | | There needs to be more support in place for partners of women who experience perinatal mental illnesses because currently it is extremely limited.  Partners are the ones who are at home with women and they need support to be able to support the new mother and baby |
|  |  |  | … interventions for partners … |
|  |  |  | Impact on further pregnancies and how to support (...) partners through it |
|  |  |  | Are all care providers trained in (...) how to support/signpost (...) partners |
|  |  |  | Supporting the mental health of (...) fathers whose babies end up needing extra support following birth |
| E | Health outcomes (women and birthing people) | | … mental health & therefore the impact of public policy on (...) mother health outcomes |
|  |  |  | Impact of birth trauma on parental wellbeing (...) the mother |
|  |  |  | Perinatal mental health impact in people (...) when their baby is in NICU and life after NICU |
| E | Health outcomes (partners) | | Impact of birth trauma on parental wellbeing (...) her partner |
|  |  |  | Perinatal mental health impact in (...) their partners when their baby is in NICU and life after NICU |
|  |  |  | … the stress of the pregnancy had essentially destroyed (...) mental health (...) of my partner … |
|  |  |  | Birth trauma and how it affects both partners |
| E | Health outcomes (baby) | | mental health & therefore impact of public policy on (...) baby (...) health outcomes |
|  |  |  | How parents’ relationships can affect the mental health during pregnancy and how this therefore affects the baby |
|  |  |  | Does autistic meltdown in pregnancy affect baby |
|  |  |  | How the birth experience effect mental health and what effects this has on baby, eg assisted delivery vs vaginal birth vs c section |
|  |  |  | Prevalence of resources and activities available in an area to attend and use during pregnancy and maternity and how that links to long term mental health and baby outcomes. |
| E | Social and economic outcomes | | NA |
| E | Parent-infant relationship | | Impact of birth trauma on parental wellbeing (both the mother and her partner) and parent-child bonding.  - Impact of baby sleep and or crying (purple crying/ colic crying) on parental mental health, parenting style and bonding to infant |
|  |  |  | Bonding between parents and child especially in a situation such as a pandemic or large crisis such as war |
|  |  |  | … Healthy parental attachment |
|  |  |  | … how that *(n.b. baby health concerns)* affects behaviours in terms of caring for your newborn |
| E | Parent-parent relationship | | … parent-parent relationship after birth |
|  |  |  | … impact on parent-parent relationships … |
|  |  |  | Partner to partner relationships and diagnosis of this |
| New | Healthcare experiences* | | … support whilst being treated |
|  |  |  | The influence of healthcare delivery - simple factors such as clinic environment/timing can make a huge difference to individuals' willingness to raise problems. These are potentially highly modifiable as well so research could be really impactful. |
|  |  |  | Postnatal care and mental wellbeing |
|  |  |  | How the quality of medical care affects likelihood of developing perinatal health difficulties. |
|  |  |  | Attitudes of health professionals towards perinatal mental health and how they conceptualise this |
|  |  |  | How midwives discuss mental health with patients from first contact, and how GPs can support |
|  |  |  | How being treated during pregnancy can affect you in regards to worries or issues that come up within your pregnancy. The treatment of hospital staff towards you during labour |
|  |  |  | The effect of neurodivergence on maternity care and pregnancy |
|  |  |  | How a midwife's work style affect a pregnant woman's experiences. |
|  |  |  | The impact of continuous care from a midwife on mental health. The links between perinatal mental health and postnatal depression/anxiety and how the transition between these can be supported. Trauma informed care training for maternity staff (including healthcare assistants who with low staffing issues are the most seen face now). |
|  |  |  | I wonder if it is possible to look at the impact of consultations and healthcare professional interactions and how that affects people’s maternity experiences and mental health after pregnancy |
|  |  |  | Gaining informed consent and communication styles. |
|  |  |  | The attitude towards mental health from professionals … |
|  |  |  | … impact of birthplace on mental health |
|  |  |  | … trauma informed care within maternity settings would have so much value - not only in regards to trauma survivors but as these principals would likely also allow for more culturally sensitive care and better care for neurodiverse individual. |
|  |  |  | …  how to avoid causing it *(n.b. trauma)* in the first place. Eg healthcare provider attitude towards birthing parents |
|  |  |  | How does neurodivergence (diagnosed/not) impact experience of pregnancy / giving birth particularly re interactions with HCPs. |
|  |  |  | How the consistency of advice and care in pregnancy affects mental health … |
|  |  |  | Hospital care of Neurodivergent patients |
|  |  |  | The impact of (...) advice given by health professionals |
|  |  |  | Training for HCPs about neurodivergent patients |
|  |  |  | Are all care providers trained in identifying signs of mental health problems and how to support/signpost birthing individuals/their partners? How do results differ in support when times are busier? E.g. do women feel more positive when staffing levels are higher? |
|  |  |  | It would be good to see how responses differ across the country. Some hospitals had 12 hours of visiting, some had 0. … |
|  |  |  | Awareness of issues and impact of interactions by clinical staff |
|  |  |  | … Comparison of how both groups *(n.b. BAME and non-BAME)* are supported by the healthcare system with respect to their mental health. |
|  |  |  | How NHS care impacts the mental wellbeing of mothers … |
|  |  |  | How body size impacts how you are treated by medical professionals during pregnancy and how this impacts mental health. |
| New | Trauma | | … the hardest and most traumatic part for me was my experience of the postnatal ward - it would be useful to include this in your work. |
|  |  |  | The role of healthcare professionals in creating birth trauma |
|  |  |  | Achieving a positive birth following birth trauma. |
|  |  |  | Cptsd … |
|  |  |  | There needs to be more protection in order to avoid trauma during hospital stays/Birthing experience |
|  |  |  | … I had a history of PTSD (...)  I was worried how the pregnancy would affect my mental health and that I could become very unwell again. I was scared of the physical examinations and labour and an internal scan triggered horrible flashbacks and could not be completed in the end. |
|  |  |  | Previous traumatic births effects on (...) mum (...) mental health |
|  |  |  | Impacts of traumatic births |
|  |  |  | Why is it some women who experience traumatic pregnancies/labour do not end up with  perinatal mental difficulties. |
|  |  |  | Trauma informed care training for maternity staff (including healthcare assistants who with low staffing issues are the most seen face now). |
|  |  |  | Impact of birth trauma on parental wellbeing … |
|  |  |  | Through my experiences I have reflected on one thing which I feel has made my pregnancy and miscarriage experiences more traumatic. I can point to a number of occasions where I wasn’t listened to and could have been offered help and support earlier. Those moments stick with me today. On the flip side there were moments where people made hard experiences much more bearable because of the time they took to explain and listen to me (this wasn’t more than 5 mins max). Those times do not stick out as traumatic |
|  |  |  | You should focus on how much parents mental health is affected by the birth. It is the most traumatising experience someone can go through if it doesn't go well. It can impact for years. |
|  |  |  | This may link with intervention but thinking about trauma informed care within maternity settings would have so much value - not only in regards to trauma survivors but as these principals would likely also allow for more culturally sensitive care and better care for neurodiverse individual. |
|  |  |  | Trauma caused by bad birthing experiences and how to avoid causing it in the first place |
|  |  |  | … And things like the 1-10 pain question just doesn’t make sense to me - and I think I had to go through more pain & trauma because of this. |
|  |  |  | … Have u gotten over birth trauma |
|  |  |  | Trauma of birth |
|  |  |  | … birth trauma … |
|  |  |  | Ptsd from birth trauma |
|  |  |  | … PTSD after traumatic birth |
|  |  |  | The effects of traumatic birth |
|  |  |  | Previous trauma not necessarily related to pregnancy or birth |
|  |  |  | The impact of traumatic births on future pregnancies … |
|  |  |  | Birth trauma and how it affects both partners |
|  |  |  | … I am desperately looking for ways to treat my baby's,  now toddler, trauma of birth … |
|  |  |  | Traumatic labour and delivery effects on mental health |
| New | Birth experiences | | The birth is important … |
|  |  |  | negative and positive influences of birth experiences … |
|  |  |  | Mental health of the mum after labour |
|  |  |  | … a parents perinatal mental health (...) for those who struggle due to 2/3/4 etc births |
|  |  |  | How did u feel about it not being ur birth plan |
|  |  |  | Benefits of elective c sections - leading to thoughts on whether they could be use more or discouraged less to protect mental health / whether benefits in mental health can outweigh some of the risks/issues to physical health. I had an elective c section due to issues with first pregnancy but I feel incredibly lucky to have had one and to know from conception I would need one. My understanding is that my recovery went so smoothly because of this. Birth was less of an unknown so I was less anxious than other pregnant people. Outcomes are generally better than for emergency c sections which many many women have despite having other plans. I'm also concerned with the mental health problems hypnobirthing courses and books can lead to as a particular birth type tends to be sold as best and it is pushed that it is down to the efforts and preparation the birthing parent puts in to achieve that. They avoid discussion of or downplay the fact that things around births are sometimes out of our hands which can lead to parents feeling like failures or as if they haven't done it right. |
|  |  |  | How the birth experience effect mental health and what effects this has on baby, eg assisted delivery vs vaginal birth vs c section |
|  |  |  | Mental health recovery of birth tears, postpartum hemmorage, long hospital and at home recovery’s. More help for those in severe pain post birth |
|  |  |  | Why there is such an increase in assisted births … |
|  |  |  | Why there is no support to get over birthing experience |
|  |  |  | Mental health issues caused by birth injury |
|  |  |  | The birth is a very special moment, is nor like any other " medical" event. A mother will always remember what she experienced during birth and everything that happens influences her. No matter what her mental wellbeing was before birth.  The birth can either give the mother incredible powers or take power and strength from her. I think that post natal depression is very connected to the birth experience. Also we should focus on baby's wellbeing and how the birth affects his consciousness and subconscious wellbeing. I am desperately looking for ways to treat my baby's,  now toddler, trauma of birth. (...)  Births should be sacred again, babies should feel safe anytime and this will bring us a better and safer society in the future. |
| New | Neurodiversity | | I would be interested in understanding more about the experiences of women with autism as well as those who have a child diagnosed with ASD |
|  |  |  | How to improve recognition of potentially higher risk individuals, and those that may be neurodiverse and not know it. Maybe have some sort of screening tool that can highlight potential conditions such as ADHD and ASD that increase the risk of adverse mental health in an otherwise seemingly low-risk person, especially since conditions like these can make it more difficult for someone to recognise they need help and actually seek out help (even more so if we throw RSD in the mix, too). I feel in my case I presented as someone who was on paper very low risk, so shortly after birth (a week or two) I was discharged. Unfortunately when they visited to check in I had my mother in law staying and felt I couldn’t be open about how I really felt, but never got another opportunity. Even if I had, it was within 2 weeks of birth, so it would have been easy to put down to hormones or baby blues. With the onus being on me to reach out if needed, things just spiralled because I didn’t feel like I could. If I’d have just had a face-to-face appointment with a postnatal doctor/healthcare professional that was dedicated to how I was doing (without it being a joint appointment with the baby), maybe a year on from birth, things might have gone differently. Or if I’d have been screened for other issues during pregnancy (especially considering my medical history, when taken as a whole, had quite a few warning signs) then maybe I’d have been offered different support, rather than just being left. I should also point out I did have a couple of unrelated appointments last year where the person doing the appointment flagged potential depression to my GP (unknown to me until I went through my history when looking into potential ADHD) and yet I’d have never known despite it being raised on two separate occasions by two different places - nobody has been touch to check in, and it just seems like nobody from the GP practice has even seen it. Sorry for going a bit off topic for the question, I just feel like I would have had a better time had I known about my neurodevelopment issues before giving birth, and it would be nice if the process could be improved to catch others like me, who come across as not needing the extra support, but actually really do later down the line, when everyone has forgotten about you. |
|  |  |  | Autism and perinatal mental health |
|  |  |  | The importance of support for identifying neurodiversity … |
|  |  |  | There wasn't a focus on neurodiversity in the research priorities: how better to support neurodiverse parents. Research relating to neurodiversity should be coproduced with those with lived experience. |
|  |  |  | Supporting neurodivergent people better in pregnancy and labour |
|  |  |  | Understanding of neurodivergence has increased so much in recent years. I feel it’s really important that this new understanding is applied in the context of perinatal mental health, so that people can be identified and diagnosed accurately. |
|  |  |  | The effect of neurodivergence on maternity care and pregnancy |
|  |  |  | research on support for autistic people particularly surrounding pregnancy and parenthood. i found that it was really difficult to get any specific support, and anything i tried to find out about only led me to things about ‘how to prevent autism during pregancy’ |
|  |  |  | Autism in pregnant women … |
|  |  |  | … better care for neurodiverse individual |
|  |  |  | Any link to Hyperemesis and Neurodiversity? |
|  |  |  | … neurodiversity and pregnancy |
|  |  |  | How does neurodivergence (diagnosed/not) impact experience of pregnancy / giving birth particularly re interactions with HCPs. I felt that the process is tailored to neurotypical people and left me feeling anxious because I felt there was a lot of stuff I was expected to know / do without being told. And things like the 1-10 pain question just doesn’t make sense to me - and I think I had to go through more pain & trauma because of this. |
|  |  |  | Hospital care of Neurodivergent patients |
|  |  |  | Support and diagnosis of mental health or neurodiversity in the initial postpartum period |
|  |  |  | Does autistic meltdown in pregnancy affect baby |
|  |  |  | … the specific struggles of autistic of ADHD birthing people. Our struggles are different and/or more extreme than neurotypicals and there is very little information out there on what to expect or how to cope, the majority of information relates to neurotypicals |
|  |  |  | It seems that people who are neurodivergent but weren't diagnosed as a child can often find out about them with the additional stresses of childcare (perhaps because they can't maintain the masking with children too!). Would adding some screening questions to the booking visit questionnaire that all pregnant women do at their first appointment have any beneficial affect? |
|  |  |  | Training for HCPs about neurodivergent patients |
|  |  |  | The relationship between different neurotypes and support available if it is given is it effective if not what can we do to support various neurotypes |
|  |  |  | Qualitative research into what experiences of perinatal mh difficulties look like for ND people. |
|  |  |  | How perinatal mental health difficulties intersect with race/ethnicity - looking specifically at the lived experiences of neurodivergent black British caribbean women. It’s something I would really like to hear more about. |
| New | Access to support/services/information/interventions | | … access to children’s centres etc. (...) access to lactation consultants and feeding community groups (LLL). |
|  |  |  | The link between perinatal mental health difficulties, and systemic inequalities (eg. As a working parent on funded maternity leave I could access la leche league, nct and children’s centers supports online) - had I been sharing a device for my children to access schooling, the impact of my distress would have been much more significant. Contextual resources don’t protect but can support MH. A further example is that women are often excluded from accessing therapy if they have no childcare for their children, perinatal services are the exception, but the threshold for entry is high. The question should be not only what support is available, but who is it offered to, is it accessible, and is what is offered sufficient. |
|  |  |  | If I’d have been screened (...) maybe I’d have been offered different support, rather than just being left |
|  |  |  | Access to information regarding birth and support … |
|  |  |  | The long queues for support. Iv waited 9 months for pregnancy support but haven't received it it will now be post natal suppport I receive. |
|  |  |  | More public information about postpartum stage |
|  |  |  | ACCESS to mental health support. Pointless studying it if no one actually gets offered it. (...)  I asked for mental health support to safeguard me and my child and hopefully make it possible for me to access maternity care and have a natural labour. They refused to see me as not actively suicidal and the clinical psychology team in primary care I was referred to urgently did not offer me an appointment until four weeks after giving birth. So pretty terrible... |
|  |  |  | Types of support available |
|  |  |  | How to access help more effectively and in a timely manner. How to not to put more pressure on the one seeking help, could GPs reach out to assess difficulties soon after birth |
|  |  |  | Long term outcomes when opportunities for intervention are missed (either accidentally or through long waiting lists) |
|  |  |  | Access to interventions for mental health and wellbeing support such as wait lists etc |
|  |  |  | Providing effective help quickly. I had to wait two months … |
|  |  |  | … I can point to a number of occasions where I wasn’t listened to and could have been offered help and support earlier … |
|  |  |  | Follow ups for people experiencing pregnancy following a loss |
|  |  |  | i found that it was really difficult to get any specific support, and anything i tried to find out about only led me to things about ‘how to prevent autism during pregancy’ |
|  |  |  | … The difficulties in accessing support when anxiety can be an overwhelming factor in seeking help |
|  |  |  | The support offered and known about by expectant parents for mental health … |
|  |  |  | What availability of support is there for mental health issues during pregnancy |
|  |  |  | …  I felt there was a lot of stuff I was expected to know / do without being told … |
|  |  |  | … I can say that the waiting list in my area for perinatal care seems longer than pregnancy. |
|  |  |  | … knowing where to get help … |
|  |  |  | Knowing where to turn if suffering during pregnancy. I was referred only by voicing concerns to a nutritionist. My midwife did not refer me when I raised them |
|  |  |  | … . Also how to get support pre pregnancy and early pregnancy as Gp receptionists all said pregnancy was nothing to do with the Gp you have to self refer to midwives but how do you do that when this is your first time you don’t know Gp staff didn’t know then when you do get in touch they say to send in your paper work at 10-12 weeks but no access to any support before that |
|  |  |  | What support is currently available to pregnant people with mental health difficulties and how do healthcare providers ensure this is actioned, and that people do not fall through the cracks in the system? |
|  |  |  | Whether socio-economic issues impact the rate of interventions in pregnancy and birth. |
|  |  |  | The availability of mental health support for first time mums - I had an incredible experience with services after giving birth to my second child, however this was down to the medications and interventions I had required postnatally after having my first son. My friend going through a first time pregnancy has not had the same experience, despite struggling with anxiety throughout |
|  |  |  | Ways to let more people know how common and NORMAL it is to experience mental issues during and after pregnancy for both parents |
|  |  |  | … mental health resources are patchy and inconsistent. There is a focus on risk management (ie risk of harm to mother and baby) which is very important but there is actually very limited support for women once it is established that their risk is low (...) quite limited real support/treatment options/follow up once a problem is identified |
|  |  |  | The complete lack of any support for people who are considered "not at risk" because they don't have the usual socio-economic factors. (...) When I asked for help I was told that I didn't qualify for any support because I wasn't self-harming or suicidal and that if I wanted therapy I could pay for it myself. I wanted practical support like what's offered by Home Start but wasn't allowed this because of my postcode. |
|  |  |  | … there is very little information out there on what to expect or how to cope, the majority of information relates to neurotypicals |
|  |  |  | not everyone is offered/aware of support. Or feel able.to accept or seek support Why is this? |
|  |  |  | The relationship between different neurotypes and support available … |
|  |  |  | It would be good to see how responses differ across the country (...) Some perinatal services only cover up to 12 months and others 24 months |
|  |  |  | (the lack of) emotional support given to people who suffer a miscarriage in the early weeks of pregnancy (before 2 months) |
|  |  |  | I long term rather than short term approaches to mental health are useful.  The difference between being seen 6 times in 6 weeks vs 6 times in 6 months.  By the time folk are seen for mental health they maybe highly motivated to do homework and therfore longer term support might work better than intensive cbt with zero follow up. |
|  |  |  | … There is so little support on the NHS. Charities pick up the shortfall in care … |
|  |  |  | … The link between lack of resources / discussion and health outcomes of individuals |
|  |  |  | Exercise support so you feel safe to exercise even after previous miscarriages.  Why there is no support to get over birthing experience. Common feelings aren’t necessarily ok feelings. |
|  |  |  | Access to treatment - waiting times, timings of diagnoses, sufficient resource within mental health services to accommodate women who need help, socio-economic barriers to accessing support. My experience has been that accessing support both after my first birth and during this second pregnancy has been incredibly difficult. It’s almost impossible to navigate the system and understand where to get help. I am incredibly privileged in terms of my socio-economic status and have very supportive friends and family. If I’m finding it challenging to access support then I can’t imagine how difficult it is for women who are in less fortunate positions than me. Support should be available to everyone who needs it and simple to navigate so that women don’t fall through the net. |
|  |  |  | How the NHS can adequately provide support for mothers having babies so that everyone can experience the same quality of care some are fortunate enough to be able to pay to access. |
|  |  |  | Prevalence of resources and activities available in an area to attend and use during pregnancy and maternity and how that links to long term mental health and baby outcomes.  My sister has a baby at start of covid and the difference in our experiences are huge. |
|  |  |  | … why people don’t access support and how to change this |
|  |  |  | Making support more easy to access - for example phone lines |
| New | Socio-economic circumstances | | Impact of social & economic outcomes on mental health & therefore the impact of public policy on baby and mother health outcomes |
|  |  |  | perhaps look at how wider family history and social status affects perinatal mental health and outcomes. |
|  |  |  | The link between perinatal mental health difficulties, and systemic inequalities (eg. As a working parent on funded maternity leave I could access la leche league, nct and children’s centers supports online) - had I been sharing a device for my children to access schooling, the impact of my distress would have been much more significant (...)  The question should be not only what support is available, but who is it offered to, is it accessible, and is what is offered sufficient. |
|  |  |  | Access to information regarding birth and support for lower income families? |
|  |  |  | Socio economic (...) risk factors for developing perinatal mental health difficulties |
|  |  |  | How low maternity pay impacts mental health |
|  |  |  | … maybe the financial implications of having a child in the UK today and how this might affect how able parents are to look after themselves well as well as their children. I feel like there’s a lot of well-being things I used to do that I had to stop for financial reasons. |
|  |  |  | Whether socio-economic issues impact the rate of interventions in pregnancy and birth. |
|  |  |  | … The socioeconomic aspect … |
|  |  |  | The complete lack of any support for people who are considered "not at risk" because they don't have the usual socio-economic factors.  (...) When I asked for help I was told that (...) if I wanted therapy I could pay for it myself. I wanted practical support like what's offered by Home Start but wasn't allowed this because of my postcode. |
|  |  |  | … Talking financial situation |
|  |  |  | … socio-economic barriers to accessing support (...) I am incredibly privileged in terms of my socio-economic status and have very supportive friends and family. If I’m finding it challenging to access support then I can’t imagine how difficult it is for women who are in less fortunate positions than me. |
|  |  |  | How the NHS can adequately provide support for mothers having babies so that everyone can experience the same quality of care some are fortunate enough to be able to pay to access. |
| New | Breastfeeding | | Breast feeding and access to lactation consultants and feeding community groups (LLL) |
|  |  |  | Impact of difficulties with breastfeeding on post-partum mood/anxiety … |
|  |  |  | Breastfeeding support |
|  |  |  | How ending breastfeeding affects mental health |
|  |  |  | Breast feeding and how promotiong it can support mental health for both mother and baby |
|  |  |  | Why there is such (...) decrease in breast feeding |
|  |  |  | Breastfeeding and mental health |
|  |  |  | … Especially support for breastfeeding and the affect it has on your mental health as you feel so pressured into doing it but not actually given any support for your mental health part of it. I've looked into it alot now about the awful feelings I got on let down of milk and its quite common but not spoken about…. |
| New | Hormones | | The profound effect of hormones post birthing |
|  |  |  | Understanding connections between certain weeks/trimesters and mental health symptoms, for example anxiety in trimester one due to rising hormone levels |
|  |  |  | How hormones affect the brain and change your responses to situations. What, why. What can you do. … |
| New | Workplace | | I work as a (*n.b. redacted*). I had to stop because each stressful interaction i had lead to heavy bleeding and problems with my pregnancy. Talking with colleagues many have had similar issues with pregnancy and many miscarried, attributing the loss of their child to the stress of their job. Research on how stressful people facing roles, like social workers, Police officers, frontline health care workers etc impacts parental and baby health |
|  |  |  | How employers (...) can work with families by adopting sensible policy |
|  |  |  | How employers support women with severe sickness and fatigue during pregnancy. How women manage this whilst working e.g whether or not to disclose before 12 weeks, what happens when sickness is only a part of the day so would prefer not to be signed off sick all together but need accommodations made. |
|  |  |  | Impact of work and stress onto pregnancy |
|  |  |  | How the workplace can react or support those struggling |
| New | Generic:  risk | | what makes vulnerable patients re-lapse |
|  |  |  | … whether or not internal or external risk factors are more important / how they interact |
|  |  |  | Any risk factors |
|  |  |  | What is the impact of living with a disability or multiple disabilities on pregnancy … |
|  |  |  | … other risk factors for developing perinatal mental health difficulties |
|  |  |  | Stress levels during pregnancy and mental health for birthing parent |
|  |  |  | Impact of (...) stress onto pregnancy |
| New | Generic: prenatal mental health | | Mental health during pregnancy |
|  |  |  | I think pre natal depression needs to be discussed in more detail. The anxiety and loneliness i felt beforehand was more so than what i felt afterwards. And I don't think this is discussed enough. |
| New | Generic: postnatal mental health | | How mental health can change after pregnancy |
|  |  |  | Postnatal mental health |
|  |  |  | Mental health after birth |
|  |  |  | Mental health of the mum after labour |
|  |  |  | How do first time parents cope in the first three months following birth? |
|  |  |  | Post baby mental health |
|  |  |  | Those first 6 weeks after a baby. Your head is a mess … |
| New | Generic: partners’ health and experiences | | Partners’ health throughout the perinatal period |
|  |  |  | Expectations of parenthood and the impact on (...) partner |
|  |  |  | Previous traumatic births effects on (...) dads mental health |
|  |  |  | Ask about how partners would rate birth and labour |
|  |  |  | More consideration to fathers |
|  |  |  | What makes partners freak out and leave their pregnant partner, particularly when this is a planned for pregnancy |
|  |  |  | How NHS care impacts the mental wellbeing of (...) partners |
| New | Generic: other relationships | | How obstetric violence affect (...) their families |
|  |  |  | … the effect of these difficulties on other relationships |
|  |  |  | Impact on other children and the wider family |
|  |  |  | How a parents perinatal mental health may affect other children for those who struggle due to 2/3/4 etc births |
|  |  |  | Is there a link between mental health difficulties a parent faces and those of future children. |
|  |  |  | Relationships and how they are impacted by pregnancy |
|  |  |  | The affect on other children in the family would be interesting although obviously not as important as others |
|  |  |  | Relationships around the mother. How relationships change with motherhood … |
| New | Comments that do not fit under a theme | | Intrusive thoughts |
|  |  |  | Post partum health |
|  |  |  | Impact on future pregnancies |
|  |  |  | Previous traumatic births effects on baby … |
|  |  |  | What is the impact of living with a disability or multiple disabilities on (...) early childhood |
|  |  |  | I think asking about how it impacted on significant others |
|  |  |  | Impact on further pregnancies … |
|  |  |  | Why do people not seek medical help and how can this be improved? |
|  |  |  | I was unsure if this study was just looking at mental health during pregnancy or postnatal mental health also, my answers would have been different if including the post natal period. |
|  |  |  | Whether the symptoms and impact of pregnancy are well understood by people in general |
|  |  |  | Reasons why people want a child |
|  |  |  | Impact of prematurity/ nicu on mental health of babies |
|  |  |  | The period immediately after birth |
|  |  |  | I found the last two questions a little confusing to answer - maybe the financial implications of having a child in the UK today and how this might affect how able parents are to look after themselves well as well as their children. I feel like there’s a lot of well-being things I used to do that I had to stop for financial reasons. |
|  |  |  | … matrescence (developmental phase of becoming a mother)... |
|  |  |  | … if there is a biological effect on the baby if their mother exercised during pregnancy |
|  |  |  | non conformist personalities and how they do or don’t fit in |
|  |  |  | … diversity is the next most important I believe and how we can create a specific separate area for mental health from before conception, during and post in regards to pregnancy |
|  |  |  | Infant impact by watching cell phone |
|  |  |  | … Research into what are the factors influencing whether baby sleeps well or is a frequent night waker … Solid research outlining that sleep issues in the first year can be rooted in factors such as prematurity, biology and individuality, and dissemination of knowledge around wide range of baby sleep needs (as Possums, BASIS research projects and Sarah Ockwell Smith as examples have already started to do) would help address this. |
|  |  |  | … Also we should focus on baby's wellbeing and how the birth affects his consciousness and subconscious wellbeing. I am desperately looking for ways to treat my baby's,  now toddler, trauma of birth … |
|  |  |  | Mental health in planned versus unplanned pregnancies |

Colours - pink=NDC, blue=no NDC, yellow=Maybe NDC

*non-directional=cannot be clearly identified as either a risk or a protective factor

E = Existing theme (i.e., Themes that match those presented as part of the quantitative ranking question, presented in the priority order as per Table 1

(Please note that some themes were subdivided into additional codes where deemed necessary – these are indicated with brackets)
